# A Neural Mechanism of Preference Shifting Under Zero Price Condition

**DOI:** 10.3389/fnhum.2016.00177

**Published:** 2016-04-20

**Authors:** Mikhail Votinov, Toshihiko Aso, Hidenao Fukuyama, Tatsuya Mima

**Affiliations:** ^1^Human Brain Research Center, Kyoto University Graduate School of MedicineKyoto, Japan; ^2^Department of Psychiatry, Psychotherapy and Psychosomatics, Medical School, Rheinisch-Westfälische Technische Hochschule Aachen, Aachen UniversityAachen, Germany; ^3^Institute of Neuroscience and Medicine (INM-6), Research Center JuelichJuelich, Germany; ^4^The Graduate School of Core Ethics and Frontier Sciences, Ritsumeikan UniversityKyoto, Japan

**Keywords:** zero-price effect, fMRI, decision-making, MPFC, neuroeconomics

## Abstract

In everyday life, free products have a strong appeal to us, even if we do not need them. Behavioral studies demonstrated that people have a tendency to switch their preference from preferred more expensive products to less preferable, cheaper alternatives, when the cheaper option becomes free. However, the neural representation of this behavioral anomaly called “Zero price” is still unclear. Using fMRI, we studied subjects while they performed binary preference choice task for items with different prices. We found that zero-related change of preference was associated with activation of the choice network, which includes inferior parietal lobule (IPL), posterior cingulate cortex and medial prefrontal cortex. Moreover, the amount of activation in medial prefrontal cortex was positively correlated with the subjective happiness score of getting free products. Our findings suggest that the Zero-price effect is driven by affective evaluations during decision-making.

## Introduction

Suppose you finish an excellent dinner at a nice restaurant. The waiter appears with a large plate full of desserts and says, “Today is a special day. You can get any one of them for free”. Even if your stomach is full, can you resist it? To get something for free has a very special effect on us, human beings. In accordance with these everyday experiences, recent studies consistently demonstrated that people respond to free products far more enthusiastically and tend to change their preference from preferred more expensive products to less preferable, cheaper alternatives, when the cheaper option becomes free (Shampanier et al., 2007; Nicolau and Sellers, 2012; Nicolau, 2012). Although results from behavioral studies demonstrated that the zero-price effect is unique, it is currently unknown which neural mechanisms underlie such switches of preferences driven by the zero-price effect.

Shampanier et al. (2007) demonstrated that the zero-price effect was linked to affect such that options with no downside (no cost) invoked a more positive emotional response and consumers used this affect as an input for their decision-making process. In addition, the decision to choose the zero-price product is simple and minimally demanding regarding computational costs (computational evaluation).

However, there is still no consensus in economic literature about which method is better in explaining consumer’s demand. The theory of revealed preferences (TRP) proposed by Samuelson (1938, 1948) suggests that consumers’ preferences could be revealed by what they purchase under different income and price circumstances. Therefore, it entails that if a consumer purchases a specific item, then that item is “revealed preferred” (Samuelson, 1938, 1948). However, there are several critical points of this model raised by other authors (Sen, 1973; Axelrod and Hamilton, 1981). TRP has been mainly criticized for not taking into account: (a) that individuals do not maintain the same value over time; (b) that preference is considered to be revealed from a single act of choice; and (c) that preferences cannot at the same time correspond to choices and personal welfare (the best example being the prisoner’s dilemma). Nevertheless we will still use term “preference” in article in line with previous research on this topic.

Positive affective evaluation and the representation of subjective value are associated with increased activity blood-oxygenation-level dependent (BOLD) in the medial prefrontal cortex (MPFC), ventromedial prefrontal cortex (VMPFC) and ventral striatum (VS), which are parts of the dopamine mesolimbic system. The MPFC region is also referred to as VMPFC or pregenual anterior cingulate cortex (pACC), but we will refer to it here as MPFC. A meta-analysis of human prefrontal activation suggested that there is a segregation of affective and cognitive functions between the medial and lateral parts of the PFC: Medial PFC was activated by emotion induction tasks, lateral PFC was recruited in cognitive tasks (Steele and Lawrie, 2004). Numerous fMRI studies reported MPFC activation in response to immediate vs. delayed reward (McClure et al., 2004a, 2007), during preference judgments (Paulus and Frank, 2003; McClure et al., 2004b), decisions during affective choice (Piech et al., 2010), favorability of brand (Ariely and Berns, 2010; Plassmann et al., 2012) and in response to the emotional salience of contents (Goel and Dolan, 2003).

While the VS is usually linked to reward processing, VS reactivity has recently been linked to stress-related anhedonia (Corral-Frias et al., 2015) and emotional numbing (Felmingham et al., 2014) in clinical populations. Furthermore, studies with healthy controls revealed that the striatum was associated with emotional responses during competitions and social comparisons (Dvash et al., 2010; Cikara et al., 2011; Votinov et al., 2015). Due to the role of these brain regions in subjective evaluation, reward and emotion processing, we hypothesize that they will be involved in preference shifting.

In addition, we predict activation in the parietal lobe, because animal and human studies revealed a general role of the parietal cortex in numerical representation (Sawamura et al., 2002; Nieder, 2005; Nieder and Dehaene, 2009). The only evidence specifically about how the concept of zero is represented in the brain comes from animal research. Okuyama et al. (2015) demonstrated that a group of neurons in the ventral intraparietal area (VIP) of the monkey was activated selectively in response to “zero” numerosity.

We rationalized that when participants need to choose between expensive, highly preferred products and cheap alternatives, the demand will be higher for the former. However, in the condition where the same cheap alternatives become free, the preferences will be switched and demand will be higher for the latter. Moreover, we hypothesized that if preference shifting is caused by integrating positive emotions into subjective valuation of zero price items, we should find specific regional activation in the MPFC, VS and parietal cortex when people choose a zero-price product.

## Materials and Methods

### Participants

All participants were recruited by announcing the study in the Kyoto University community and all were paid for their participation in this experiment. Participants were excluded for any of the following conditions: MRI contraindications, psychological or neurological pathology, a history of seizures, suspected pregnancy and claustrophobia. Fourteen healthy participants (three females) with normal or corrected-to normal vision took part in the study. Due to a lack of compliance (one subject did not understand the task and one had excessive motion) and technical problems (scanner crashed during measurement), three participants were excluded from the analyses. The average age of the remaining 11 (two females) participants was 28.8 ± 6.3 years old. All were right-handed as assessed by the Edinburgh Handedness Inventory (Oldfield, 1971). The study was approved by Kyoto University Graduate School and the Faculty of Medicine Ethics Committee and conducted in accordance with the Declaration of Helsinki. All volunteers participated in the study after giving written informed consent.

### Experimental Paradigm

Participants were asked to choose between and purchase one of two products (one low-value, one high-value) from the same category with different prices, in a similar manner as reported by Shampanier et al. (2007). The product pairs were designed such that when both products are appropriately priced, people would choose the high-value product even if they have to pay more to get it.

We prepared 14 product pairs from the same category (e.g., Godiva chocolate bar and domestic brand cheap chocolate bar) of different types of goods, including food, drinks, electronic gadgets and accessories. Here is a list of all pairs: (1) The expensive chocolate brand and cheap chocolate brand; (2) Expensive mobile phone brand and cheap mobile phone brand; (3) Expensive purse and cheap purse; (4) Mug with expensive coffee and mug with cheap instant coffee; (5) E-reader and book; (6) Expensive big cake and cheap cookie; (7) Bottle of very expensive Sake (Japanese alcohol) and cheap sake; (8) Expensive dish of Ramen (Noodle) and cheap cup of instant noodle; (9) Expensive Carry rice dish and dish with just plain rice; (10) Big Hamburger set and one hamburger; (11) Expensive Udon (wheat flour noodle) with Tempura (seafood or vegetables that have been battered and deep fried) and cheap plain Udon; (12) Expensive dish with Kobe beef (known for well-marbled texture) and cheap piece of chicken; (13) Expensive salad from real crabs and cheap salad from crab sticks (imitation crab meat); and (14) Expensive soup with matsutake (very rare and expensive mushroom in Japan) and cheap instant soup.

Before the main experiment, we asked participants to make a hypothetical choice between each pair to assess their preferences by using a five points Likert scale (1—“strongly unpreferred”; 5—“strongly preferred”), and asked them to indicate a maximal amount of money that they would be willing to pay (WTP) for each product. Participants made one choice for each pair of product. The Likert scale had forward and reversed order and was balanced across the subjects. We used a forced choice, when both items were presented simultaneously and participants needed to declare which one they preferred. After the preference test, we chose eight pairs individually, where the high value product (hereafter “HP”) was more preferable than the low value product (hereafter “LP”), which were then used for the main fMRI experiment. If we had more than eight pairs we chose the pair with a higher score on the preference scale. If pairs had the same score, we flipped the coin and chose one. The individual WTP prices determined by the participants in the preliminary test were used as the initial cost for each item.

**Figure 1:**
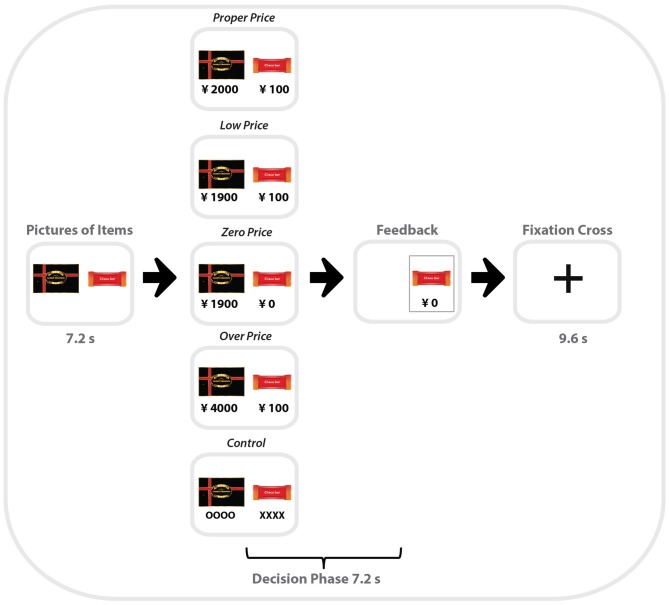
**Schematic illustration of one trial of the zero-price task performed by participants in the MRI scanner.** Middle column is example of all five types of binary choices used in the task (from top to bottom): Proper Price, Lower Price, Zero Price, Over Price and Control.

Each pair of products within one category was presented for 7.2 s (or 3 TR), followed by the choice period (7.2 s, when participants saw the prices for the two items. During the first period, participants had time to identify and compare the items, so that in the choice period, they could focus on the prices and make their decision regarding which item to choose. Participants were asked to make their choice as fast as possible by pressing left or right buttons using a non-magnetic device. When the participant pressed a button, the non-chosen item disappeared from the screen. Task blocks were separated by a resting period lasting 9.6 s with only a central fixation point presented (Figure 1).

All product pairs were arranged in the following five price conditions:

For the condition “Proper Price”, the individual maximum WTP prices determined by the participants in the preliminary test were used as the initial cost for each item. Thus, this condition represented the borderline of the participants’ willingness to pay a particular price for a particular item. HP and LP were priced as P(HP) and P(LP), respectively, e.g., 2000 Japanese yen and 100 Japanese yen. Participants mostly selected HP by paying P(HP) because HP is more preferable.

For the condition “Low Price”, HP was priced as (P(HP)−P(LP)) and LP was priced as P(LP), e.g., 1900 Japanese yen and 100 Japanese yen. We expect that subject would select HP, in the same way as the condition “Proper Price”.

For the target condition “Zero Price”, both products were discounted by P(LP). Thus HP and LP were priced as (P(HP)−P(LP)) and zero, e.g., 1900 Japanese yen and 0 Japanese yen. In spite of the same amount of discount, we expect that participants would “irrationally” change their preference and select LP because it was free.

For the condition “Over Price”, HP was priced as the double of P(HP) and LP was priced as P(LP), e.g., 4000 Japanese yen and 100 Japanese yen. We expect that participants would “rationally” change their preference and select LP, because HP was over-priced compared to its use-value.

**Figure 2:**
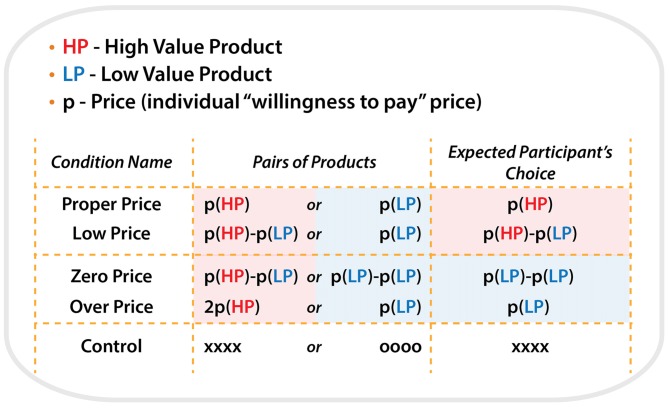
**Schematic illustration of the all types of conditions, price combinations for pairs of products during each condition and expected participant’s choices**.

For the “Control” condition, HP and LP were presented without prices. Instead of prices, “oooo” or “xxxx” were presented. Participants were asked to select xxx by pressing one of the buttons. The control condition served to control visual-motor aspects of the target experiment (Figure 2).

Each fMRI run was preceded by three dummy scans (7.2 s) allowing the MR scanner to reach a steady T2* contrast. Then, the fixation cross was presented for 9.6 s. Therefore, every 1st trial in each run started from 16.8 s and total time for each run was 520.8 s.

Each run contained four product pairs under five different price conditions and to avoid possible effects of order, all conditions were presented in a pseudorandom order for each participant. Thus participants evaluated each product pair twice throughout the experiment.

### Behavioral Data

The reaction time of the button press was measured from the price onset. Also we analyzed the proportion of participants’ demand for HP and LP across the four price conditions. After the scan session, participants filled in a questionnaire indicating on a five point Likert scale how happy they felt about each decision they had made. The behavioral results were analyzed in SPSS 20.0 (SPSS Inc., Armonk, NY, USA) using a repeated-measures ANOVA with *post hoc* comparisons with Bonferroni correction for multiple comparisons. If the sphericity assumption was violated (significant results in Mauchly’s test of sphericity), degrees of freedom were corrected using Greenhouse-Geisser estimates of sphericity. Data are reported as mean ± SEM.

### Image Acquisition and Analysis

#### MRI Data Analysis

FMRI experiments were conducted on a 3T Trio whole body scanner (Siemens, Erlangen, Germany). Functional images were obtained with a T2*-weighted gradient echo, echo-planar imaging sequence. The image acquisition parameters were as follows: repetition *time* (TR) = 2.4 s, echo *time* (TE) = 30 ms, flip *angle* (FA) = 90°. For anatomical registration, we obtained high-resolution 3D T1 anatomical images after the fMRI session (magnetization prepared rapid gradient-echo sequence, 0.94 × 0.94 × 1 mm^3^ voxel, 2 s repetition time, 4.38 ms TE, 990 ms inversion time, 8° FA, 130 Hz bandwidth). Participants lay supine on a scanner bed, with a button response device held with their right hand. They viewed visual stimuli back-projected onto a screen through a mirror. Foam pads and elastic tape were used to minimize head motion. Image analysis was performed using statistical parametric mapping software (SPM8: http://www.fil.ion.ucl.ac.uk/spm) in MATLAB (Mathworks Inc., Natick, MA, USA). The functional images were corrected for sequential slice timing and were realigned to the first image to adjust for head movements. The realigned images were then spatially normalized to a template brain (Montreal Neurological Institute, QC, Canada) provided by SPM8 (Ashburner et al., 1997). Finally, the images were smoothed with an isotropic Gaussian Kernel of 8 mm full width at half maximum (FWHM).

#### Statistical Analysis of the fMRI Data

The first level (individual subject) analyses were set up using the general linear model approach, with events of interest being modeled as regressors. The following events were modeled: items presentation periods and decision-making periods for each condition. These individual decision-making contrasts from the first-level were then taken to a second-level group analysis using an ANOVA (factor: condition, subject), thus employing a random effects model.

First, to investigate the brain areas associated with price evaluation and choice preference for each condition (“Zero Price”, “Proper Price”, “Low Price”, and “Over Price”) were compared to the Control condition.

The focus of the analyses was on the contrasts associated with the zero price in order to isolate brain activity accompanying the zero-price effect. We compared “Zero Price” and “Low Price” conditions, which would reflect the preference switch from HP to LP. In this comparison, the amount of price discount was matched, so that both chosen products were discounted by P(LP) and LP became zero price.

Next, we computed Zero Price vs. Over Price. In both conditions, the participants choose the LP, but in one condition they choose LP because it had zero price and in another condition because HP became twice expensive. In addition, we compared “Over Price” vs. “Low Price” conditions, which reflect comparison of decision to choose LP product vs. decision to choose HP, but without zero price.

To investigate the brain region specifically associated with Zero price preference, we performed a conjunction analysis for the comparison “Zero Price” > “Low Price”, “Zero Price” > “Over Price”, “Zero Price” > “Proper Price”.

For all analysis, we used a family-wise error (FWE) correction at the voxel level at a threshold of *P* < 0.05, for identifying statistically significantly activated voxels. In some cases, where we had strong *a priori* hypotheses, data were also explored at more liberal thresholds (see “Results” Section).The resulting activation maps were displayed onto the T1 template from SPM8 Software package to identify the anatomical correlates of the respective brain activity.

Additionally, we conducted exploratory multiple regression whole brain analyses to investigate the relationship between total individual happiness scores for decisions during the zero condition and activation from the other pricing conditions. The results are reported at a threshold of *P* < 0.001, uncorrected for multiple comparisons.

## Results

### Behavioral Data

Mean reaction times (RTs) were not significantly different between price evaluation conditions (*F*_(1,1.3)_ = 0.59, *P* = 0.5, *η*^2^ = 0.05); Proper Price: 2.61 ± 0.23, Low Price: 2.27 ± 0.22, Zero Price: 2.21 ± 0.32 and Over Price: 2.56 ± 0.65. While one would expect shorter RT during Zero Price, due to the simplicity of comparing zero with bigger numbers, this was not a case: the time needed to make decisions in the Zero Price condition was not significantly different than in the other conditions.

**Figure 3:**
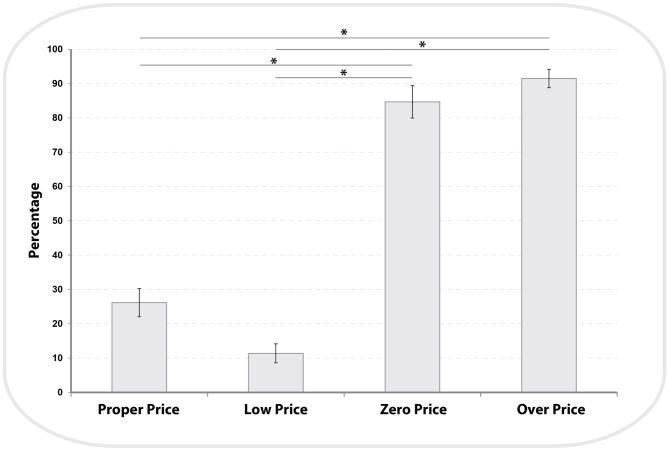
**Distribution of demand for Low Price (LP) items across all four pricing binary choices.** The demand for LP items during Zero and Over conditions was significantly higher (see*) compared to Proper and Low price conditions (see “Results” Section).

We analyzed the participants’ demand for high value products (HP) and low value products (LP) across the four price evaluation conditions (see Figure 3). There was a main effect of price conditions (*F*_(1,3)_ = 163.3, *P* < 0.001, *η*^2^ = 0.9). Bonferroni-corrected *post hoc* comparisons demonstrated that the demand for LP was significantly different between each condition (Proper Price: 26.13 ± 4.1, Low Price: 11.36 ± 2.7, Zero Price: 84 ± 4.7 and Over Price: 91 ± 2.7; all *P* < 0.002), with the exception of a non-significant difference between Zero Price and Over Price conditions (*p* = 1). These results confirmed our prediction that the demand for HP items will be higher for Proper and Low Price conditions and will be reversed for the Zero and Over Price conditions.

Regarding contentment/happiness score, there was a main effect of Task (*F*_(1,3)_ = 54.8, *P* < 0.001, partial *η*^2^ = 0.84). Bonferroni-corrected *post hoc* comparisons demonstrated that the participants were significantly happier about their choices during the “Zero price” (4.4 ± 0.09) compared to the “Proper Price” (3.08 ± 0.06, *P* < 0.001) and “Over Price” conditions (2.68 ± 0.1, *P* < 0.001). However, regardless of being happier in the “Zero Price” (4.4 ± 0.09) than in “Low Price” (3.93 ± 0.08) condition there was no significant difference between them (*p* = 0.4).

### Imaging Results

#### Price Conditions vs. Control Condition

**Figure 4:**
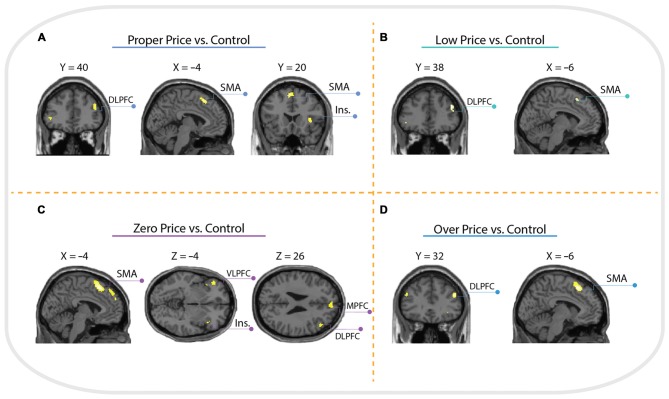
**Whole brain activation of all participants for: (A) Proper Price > Control revealed activation in insula (Ins.), supplementary motor area (SMA) and dorsolateral prefrontal cortex (DLPFC); (B) Low Price > Control revealed activation in SMA and DLPFC; (C) Zero Price > Control revealed activation in Insula, SMA, ventrolateral prefrontal cortex (VLPFC) and DLPFC; (D) Over Price > Control revealed activation in DLPFC and SMA.** The threshold is *P* < 0.05 family-wise error (FWE) corrected, at voxel level.

Brain activations associated with price evaluation tasks compared to the visual-motor control task were characterized by a similar pattern across all four conditions, with peak activity observed in the anterior insula, supplementary motor area (SMA) and dorsolateral prefrontal cortex (DLPFC, Brodmann Area 46). However, the contrast Zero Price vs. Control additionally revealed activation in left ventrolateral prefrontal cortex (VLPFC) and MPFC see (Figure 4).

#### Zero-Price Specific Analysis

To isolate activity specific to the zero-price effect, we compared the brain activation patterns during the decision-making process in the Zero Price condition against the other pricing conditions.

**Figure 5:**
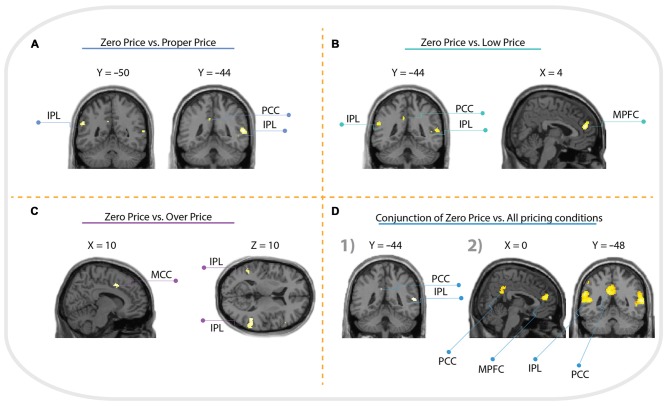
**Whole brain activation of all participants for: (A) Zero Price > Proper Price revealed activation in inferior parietal lobule (IPL) and posterior cingulate cortex (PCC); (B) Zero Price > Proper Price revealed activation in IPL, PCC and medial prefrontal cortex (MPFC); (C) Zero Price > Proper Price revealed activation in IPL, PCC and middle cingulate cortex (MCC).** The threshold is *P* < 0.05 FWE corrected, at voxel level. The conjunction analysis with the threshold is *P* < 0.05 FWE corrected at voxel level **(D1)** revealed activation in IPL, PCC; and analysis with threshold *P* < 0.05 cluster level corrected **(D2)** revealed activation in IPL, PCC and MPFC.

For the comparison “Zero Price” > “Proper Price”, we found increased activity in inferior parietal lobule (IPL) and PCC only (see Figure 5A, Table 1).

**Table 1:** **Cluster list of activation for contrast Zero Price > Proper Price (threshold *P* < 0.05 FWE corrected at voxel level) of all participants**.

Brain areas (aal)	Side	Cluster size	*x*	*y*	*z*	*T*
IPL	R	133	60	−44	10	7.06
IPL	L	51	−56	−50	26	6.70
PCC	L	14	−8	−46	34	5.96
Middle temporal gyrus	R	1	52	−42	2	5.85
Superior temporal pole	L	3	−52	10	−22	5.62
Angular Gyrus	R	10	58	−54	26	5.53
Middle temporal gyrus	R	1	50	−44	0	5.49
Middle temporal gyrus	R	1	50	−42	−4	5.38

*Note: L/R, left/right in the brain; sc, sub-cluster; PCC, posterior cingulate cortex; IPL, inferior parietal lobule*.

The most interesting contrast revealing the brain activation underlying a switch in preference was “Zero Price” > “Low Price”. We compared the condition when participants chose low value (non preferred) products over expensive alternatives against the condition when participants chose high value products over cheap alternatives. Behavioral results demonstrated a significant switch in demand from (HP) to (LP) after discounting of (LP). The fMRI analysis revealed increased activity in MPFC, IPL and PCC for this contrast (Figure 5B, Table 2). The reverse contrast did not reveal any significant activation.

**Table 2:** **Cluster list of activation for contrast Zero Price > Low Price (threshold *P* < 0.05 FWE corrected at voxel level) of all participants**.

Brain areas (aal)	Side	Cluster size	*x*	*y*	*z*	*T*
MPFC	R	145	4	50	20	7.94
IPL	L	110	−56	−50	26	7.08
IPL	L	s.c.	−56	−42	24	5.84
Superior temporal gyrus	R	47	60	−44	10	6.09
Middle temporal gyrus	L	3	−60	−4	−10	5.89
PCC	L	29	−6	−46	34	5.87
Superior temporal gyrus	R	11	60	−28	10	5.85
Superior temporal gyrus	R	s.c.	56	−22	6	5.74
Superior temporal gyrus	R	9	50	−42	8	5.74
IPL	R	5	58	−50	24	5.65
Superior temporal gyrus	L	4	−50	−50	14	5.59

*Note: L/R, left/right in the brain; s.c., sub-cluster; PCC, posterior cingulate cortex; IPL, inferior parietal lobule; MPFC, medial prefrontal cortex*.

For the comparison “Zero Price” > “Over Price”, the brain activations were observed in dorsal ACC/MCC, IPL and PCC (Figure 5C, Table 3). During both conditions participant chose the LP over HP, but the reasoning behind these decisions were different. In the former condition they chose LP, because LP became free, but in the latter because the preferred HP became twice as expensive. Thus, while significant activation differences were observed for LP becoming free (Zero price > Over price), HP becoming twice as expensive (Over price > Zero price) did not give rise to significant activation differences. As well, the comparison “Over Price” > “Low Price” and reverse did not reveal any significant differences in brain activation at the threshold FWE *P* < 0.05.

**Table 3:** **Cluster list of activation for contrast Zero Price > Over Price (threshold *P* < 0.05 FWE corrected at voxel level) of all participants**.

Brain areas (aal)	Side	Cluster size	*x*	*y*	*z*	*T*
MCC	R	68	12	12	42	6.73
IPL	R	143	48	−44	10	6.49
IPL	R	s.c.	62	−42	8	6.28
IPL	L	26	−44	−50	8	6.15
IPL	L	s.c.	−52	−52	10	5.70
Superior temporal pole	L	26	−58	−40	20	5.97
PCC	L	2	−8	−44	32	5.45
VLPFC/BA45	R	1	56	26	10	5.45

*Note: L/R, left/right in the brain; s.c., sub-cluster; MCC, medial cingulate gyrus; PCC, posterior cingulate cortex; IPL, inferior parietal lobule; VLPFC, ventrolateral prefrontal cortex*.

The conjunction analysis of Zero Price condition vs. all other conditions (Proper Price, Low Price, Over Price) revealed clusters in IPL and PCC (Figure 5D). However, the cluster in MPFC showed trend to be significant too (*p* = 0.022 cluster-level corrected, *T* = 4.86, with peak at *x* = 4, *y* = 48, *z* = 18, and extent threshold *k* = 190 voxels), see (Figure 5D). This demonstrates that all these three regions were more involved in decision-making process during Zero Price condition.

The exploratory whole brain regression analysis investigating the relationship between individual happiness scores for decisions during the Zero Price and other pricing conditions revealed a positive correlation with subjective happiness scores and activation in MPFC (*x* = −8, *y* = 48, *z* = 6, *T* = 7.52, *k* = 56, and *x* = 8, *y* = 48, *z* = 10, *T* = 5.52, *k* = 19) during Zero Condition > Low Price condition only.

## Discussion

The current study examined the neural mechanisms which underlie the switch of preferences driven by the zero-price effect. Confirming our hypothesis, behavioral data demonstrated a powerful effect of the zero price, in line with previous reports (Shampanier et al., 2007; Nicolau and Sellers, 2012; Nicolau, 2012). High value preferred products were consistently chosen in conditions in which HP and LP corresponded to the participants’ initial minimal WTP price, and in the condition in which HP was discounted by the price of LP. However, when the conditions included a free product or preferred items had double price, the preferences were reversed. Moreover, RTs for choices during different pricing conditions were not significantly different, implying that participants’ choices during the Zero Price condition were not just driven by simplicity.

The analysis of happiness ratios revealed that participants were significantly happier with their decisions regarding zero price items compared to other conditions. Overall, behavioral data demonstrated that the demand for low value products was reversed in conditions where LP had zero price and when HP had double price.

Regarding fMRI data and “Zero Price” specific analysis, the comparison between decision-making during the “Zero Price” condition vs. the “Low Price” condition revealed brain activation in MPFC, IPL and PCC regions. In contrast, the comparison of the “Zero Price” condition vs. the “Over Price” condition was also associated with increased activity in the IPL and PCC, but also in the dorsal ACC/MCC. Moreover, the conjunction analysis showed that IPL, PCC and MPFC were the “Zero Price” specific regions.

One previous animal study found that a group of neurons in the inferior parietal lobe (IPL) of monkeys was activated only in response to the numerosity “zero” (Okuyama et al., 2015). In the same vein, previous fMRI studies demonstrated that these regions are crucial for numerical processing (for a review, see Nieder and Dehaene, 2009) and number comparisons (Chiao et al., 2009). Therefore, since our data also demonstrated that the IPL area was highly involved in Zero Price condition, we suggest that the IPL in humans also plays a crucial role in the processing of zero numerosity.

The PCC is an anatomically heterogeneous region, and the cluster of activation in PCC that we observed here is located within the ventral posterior cingulate cortex (vPCC), according to the classification of 2003 (Vogt et al., 2003). Vogt et al. (2006) conducted a functional connectivity study with PET on histologically guided regions of interest in PCC, and proposed that vPCC engaged in self-reflection via connections and integration of input from subgenual ACC/MPFC. The authors concluded that the vPCC is not part of an emotion system *per se*, but provides the code for relevant information from visual sensory systems to evaluate emotional content (Vogt et al., 2006). Moreover, we also observed that vPCC was co-activated with MPFC and IPL. A recent study using meta-analytic connectivity modeling (MACM) and resting-state functional connectivity (RSFC) approaches demonstrated that, indeed, there is connectivity between vPCC, IPL and MPFC regions (Bzdok et al., 2015). Thus, it is likely that vPCC in our study plays an integrative role too.

Previous animal and human studies underlined the role of brain structures such as the MPFC/VMPFC in goal-directed decision-making; when subjects need to choose between actions that are associated with different reward outcomes and different costs. For example, in a study by Chib et al. (2009) authors investigated the neural correlates of economic decisions between consumer goods, food, and monetary rewards, and observed that a common area in VMPFC was activated during evaluation of all types of goods. A similar area was activated in the study by McClure et al. (2004b), where activation in VMPFC correlated with participants’ behavioral preferences for beverages. Moreover, Plassmann et al. (2007) found that this area encodes the participants’ willingness-to-pay (WTP) computation in which buyers calculate the maximum amount of financial resources that they are willing to give up in exchange for the object. Studies investigating decision-making during a purchase also demonstrated that activation in MPFC can predict participants’ subsequent choices (Knutson et al., 2007; Tusche et al., 2010). Altogether, these and many others findings of activation in MPFC/VMPFC shed light onto the crucial role of this area in preferences during binary choices, the representation of subjective value and guiding choices during purchasing or choosing between products. Moreover, activation in MPFC was observed in response to immediately available rewards vs. delayed reward (McClure et al., 2004a, 2007) and in decisions during affective choice (Piech et al., 2010). In the other study, the authors revealed in response to favorite brands increased activation in areas involved in the processing of emotions and self-reflection during decision making, such as the posterior cingulate (BA 7), right superior frontal gyrus (BA 10), and most pronounced, the VMPFC (BA 10; Deppe et al., 2005). The primary reward studies also demonstrated that activity in MPFC correlated with both health and taste ratings at the time of decision (Hare et al., 2010, 2011), with richness and pleasantness of touch (McCabe et al., 2008), and showed activation that was linearly related to the easiness of both olfactory and warm pleasantness choices (Rolls et al., 2010). These findings suggest that MPFC is involved in the assignment of an emotional (subjective) value to guide preference choices. Our behavioral findings demonstrated that people were significantly happier during the Zero Price condition. In addition, multiple regression analysis revealed a positive correlation between subjective happiness score and activation in the MPFC. It is thus likely that the zero price option elicits positive emotions and increases the subjective value of LP, leading to a change of preferences. This supports our assumption of affective evaluation (or rather a positive affective response) being an important mechanism driving this effect. Given that positive affective responses may underlie the zero price effect, one might expect activation in another areas related to emotional processing, such as the amygdala. Indeed, several meta-analyses (Costafreda et al., 2008; Sergerie et al., 2008; Lindquist et al., 2012) demonstrated that the amygdala responds to all visual emotional stimuli regardless of valence, and that it plays a general role in detecting socially and biologically relevant information, especially from the face expression. However, in our task the stimuli used were not emotional *per se* (images of products), and price information is not a biologically relevant information that could be related to surviving, for example. This could explain why we did not observe activation in classic emotional areas such as the amygdala during the Zero price condition.

However there are several limitations of our study. First, a small sample size. However, our results were appropriately controlled for false positives (we reported only FWE corrected results). In some cases where we had strong *a priori* hypotheses, data were also explored at more liberal thresholds. Second, there were no actual monetary incentives offered in the fMRI task, i.e., no real payments and cost outcomes were involved. Therefore, the here observed neural meaning of “cost free” and “happiness” might be slightly different compared to tasks involving actual incentives. However, Miyapuram et al. (2012) demonstrated that hypothetical monetary incentives can be reliably used to study the human reward system (Miyapuram et al., 2012). The third limitation is our happiness rating. Since this was a *post hoc* measure that consisted of only one question, it could represent not only affect/mood but also decision confidence, reduction of conflict or ease of choice. Thus, future studies are needed to replicate our findings by using larger sample sizes and by using a more sophisticated questionnaire, which takes into account measures of emotion, confidence and easiness of choice to disentangle possible effects of these factors.

Taken together, our study confirms previous behavioral findings that the zero price can change preferences regarding previously wanted items. When we compared the Zero Price condition with other pricing conditions, we observed higher activation in the choice brain circuit, including IPL, PCC and MPFC regions. We suggest that the zero price may elicit a strong positive emotional reaction, which may trigger a choice in favor of products with zero price. In conclusion, our findings indicate that positive affective responses may underlie the zero price effect.

## Author Contributions

MV, TA, HF and TM were involved in designing the study, as well as in writing and editing the manuscript. MV, TA and TM collected and analyzed the data. All authors reviewed the manuscript.

## Funding

This study was partly supported by Grant-in-Aid for Scientific Research (B) 15H03044, Grant-in-Aid for Scientific Research on Innovative Areas (15H05880, 15H05871) from the Japan Society for the Promotion of Science, 27280201 and 2736040 from Japan Agency for Medical Research and development (AMED) (to TM).

## Conflict of Interest Statement

The authors declare that the research was conducted in the absence of any commercial or financial relationships that could be construed as a potential conflict of interest.
